# Links Between the Amplitude Modulation of Low-Frequency Spontaneous Fluctuation Across Resting State Conditions and Thalamic Functional Connectivity

**DOI:** 10.3389/fnhum.2019.00199

**Published:** 2019-06-13

**Authors:** Shufang Qian, Xinbo Wang, Xiujuan Qu, Peiwen Zhang, Qiuyue Li, Ruidi Wang, Dong-Qiang Liu

**Affiliations:** Research Center of Brain and Cognitive Neuroscience, Liaoning Normal University, Dalian, China

**Keywords:** functional connectivity, amplitude of low-frequency fluctuation, thalamus, lateral geniculate nucleus, pulvinar, eyes open, eyes closed

## Abstract

A comparison of the different types of resting state reveals some interesting characteristics of spontaneous brain activity that cannot be found in a single condition. Differences in the amplitude of low-frequency fluctuation (ALFF) between the eyes open (EO) and the eyes closed (EC) almost have a spatially distinct pattern with traditional EO-EC activation within sensory systems, suggesting the divergent functional roles of ALFF and activation. However, the underlying mechanism is far from clear. Since the thalamus plays an essential role in sensory processing, one critical step toward understanding the divergences is to depict the relationships between the thalamus and the ALFF modulation in sensory regions. In this preliminary study, we examined the association between the changes of ALFF and the changes of thalamic functional connectivity (FC) between EO and EC. We focused on two visual thalamic nuclei, the lateral geniculate nucleus (LGN) and the pulvinar (Pu). FC results showed that LGN had stronger synchronization with regions in lateral but not in medial visual networks, while Pu had a weaker synchronization with auditory and sensorimotor areas during EO compared with EC. Moreover, the patterns of FC modulation exhibited considerable overlaps with the ALFF modulation, and there were significant correlations between them across subjects. Our findings support the crucial role of the thalamus in amplitude modulation of low-frequency spontaneous activity in sensory systems, and may pave the way to elucidate the mechanisms governing distinction between evoked activation and modulation of low-frequency spontaneous brain activity.

## Introduction

Accumulating evidence has suggested that spontaneous blood oxygenation level-dependent (BOLD) signal measured in the resting human brain is specifically organized (Biswal et al., 1995; Lowe et al., 1998; Cordes et al., 2000; Greicius et al., 2003; Fox and Raichle, 2007). The resting state refers to a condition that requires subjects to rest quietly without any cognitive tasks. Three types of resting conditions are often applied: fixation, eyes open (EO) and eyes closed (EC). Most studies focused on a single condition to depict the resting brain networks (Fox and Raichle, 2007; Power et al., 2011; Yeo et al., 2011), to compare functional organization between normal subjects and patients (Greicius, 2008; Fox and Greicius, 2010; Zhang and Raichle, 2010) or to study intrinsic BOLD signal variability (Zang et al., 2007; Zou et al., 2008). When comparing the regional features of BOLD signal between distinct types of resting state, some surprising results emerged. We have consistently revealed that between EO and EC, the amplitude of low-frequency fluctuation (ALFF, Zang et al., 2007) exhibited symmetrically distributed and highly reproducible differences that were located in the extrastriate areas and non-visual sensory modalities, including primary auditory cortex (PAC) and somatosensory areas (Yan et al., 2009; Liu D. et al., 2013; Yuan et al., 2014; Zou et al., 2015; Zhang et al., 2018), with little overlap with the expected primary visual cortex (PVC). Hence, spontaneous brain activity can presumably be modulated by external or internal environments in a very different way from traditional task evoked activation, which might encode their divergent functional roles.

The underlying mechanism for such distinction is far from clear. We speculated that the thalamus may be a critical region that is responsible for the modulation of regional spontaneous activity in the aforementioned sensory areas. On one hand, the observed EO-EC ALFF differences are located primarily in the sensory regions. On the other hand, the thalamus is a centrally located relay station that has tight interaction with different subcortical and cortical areas and controls all the sensory information flowing into the cortex (Sherman and Guillery, 2006; Sherman, 2007). Recent evidences have demonstrated that local processing of an area largely relies on its connectivity with other brain regions (Cole et al., 2016; Tavor et al., 2016; Ito et al., 2017), suggesting that the local activity and synchronization among brain regions could be integrated into a more unified framework. Motivated by this notion, in this preliminary study we wondered whether modulation of cortical ALFF between EO and EC is associated with thalamic functional connectivity (FC).

It is noteworthy that the function of the thalamus is heterogeneous across different nuclei. Sherman divides the thalamic relays into two types according to driver sources: first order (FO) and higher order (HO) (Sherman and Guillery, 2006; Sherman, 2016). The driver input of FO comes from subcortical areas, while HO receives a cortical driver input and represents a key link in cortico-thalamo-cortical circuits. Considering the most significant external difference between EO and EC, i.e., whether subjects receive visual input or not, we are only interested in the visual nuclei for this study. In visual system, the lateral geniculate nucleus (LGN) is the FO that represents the first relay of visual information from the retina to the cortex. The pulvinar (Pu) is HO, which participates in further cortical processing of information via transthalamic corticocortical pathway (Sherman, 2016). Although both nuclei belong to the visual system, little is known about how the FC between these thalamic relays and cortex changes across EO and EC, and more importantly, whether the changes of thalamic FC are related to the EO-EC differences in ALFF. Zou et al. (2009) paid attention to Pu’s FC across states but narrowed down the range to include the visual cortex only, which neglected other probably meaningful areas, considering Pu’s involvement in multi-sensory processing (Cappe et al., 2009; Bridge et al., 2016).

In this study, we compared thalamic FC between EO and EC for LGN and Pu, and examined their relationships with EO-EC ALFF differences. First, the effects of resting state types on LGN and Pu’s FC were examined. Second, we performed overlap and quantitative correlation analyses between FC and ALFF based EO-EC differences. Our results indicated different patterns of FC modulation for the two nuclei, as well as their respective associations with ALFF modulation in multiple sensory networks, thereby supporting the involvement of thalamus in modulating ALFF in sensory systems, which provided profound insights for further studies about the underlying mechanisms.

## Materials and Methods

### Data Acquisition

This dataset is publicly available at NITRC^1^ (Beijing: eyes open and eyes closed study) (Liu D. et al., 2013). A group of 48 participants (24 females, aged 19–31 years) were scanned using a Siemens Trio 3T scanner in the Beijing Normal University Imaging Center for Brain Research. Written informed consent was obtained from each subject. Three resting state scans were collected per subject: one EC scan (data of this scan was obtained for other purpose and was not analyzed here) and two counterbalanced scans (EO and EC in a random order). Each scan lasted for 8 min. Functional images were acquired using an echo-planar imaging (EPI) sequence with the following parameters: TR/TE = 2000/30 ms, flip angle = 90°, 33 axial slices, in-plane resolution = 64 × 64, thickness/gap = 3.5/0.7 mm, FOV = 200 mm^2^ × 200 mm^2^, 240 volumes. For the purpose of spatial normalization, a 3D T1-weighted sagittal magnetization-prepared rapid gradient echo (MP-RAGE) sequence was also acquired: TR/TE/TI = 2530/3.39/1100 ms, flip angle = 7°, 128 slices, acquisition matrix = 256 × 192, thickness/gap = 1.33/0 mm, FOV = 256 mm^2^ × 256 mm^2^. Additionally, each subject underwent a diffusion weighted imaging (DWI) scan by the single-shot EPI sequence with the following parameters: TR/TE = 7200 ms/104 ms, flip angle = 90°, 49 axial slices, acquisition matrix = 128 × 128, FOV = 230 mm^2^ × 230 mm^2^, voxel size = 1.8 mm^3^ × 1.8 mm^3^ × 2.5 mm^3^, *b*-value = 1000 s/mm^2^, 64 non-collinear directions. An image without diffusion weighting (*b* = 0) was also acquired. Three subjects were excluded due to the lack of DWI data.

### Data Preprocessing

For the functional and anatomical images, data preprocessing and subsequent analyses were performed using the Data Processing and Analysis of Brain Imaging toolbox (DPABI^2^, Yan et al., 2016). Briefly, the preprocessing steps consisted of discarding the first 10 volumes, slice-timing correction, head motion correction, spatial normalization to the Montreal Neurological Institute (MNI) template, resampling to 3 mm cubic voxels and smoothing with a Gaussian kernel of 4 mm full width at half maximum (FWHM). To verify that our results were not biased by the spatial blurring of nuclei boundaries, we also presented results without smoothing in the Supplementary Materials. Linear trends regression and temporal bandpass filtering (0.01–0.1 Hz) were then applied. For nuisance regression, several components were considered, including averaged signals within white matter (WM) and cerebrospinal fluid (CSF) masks, as well as head motion effects modeled with a Friston 24-parameter (6 head motion parameters along with their first derivatives and the 12 corresponding squared items) model (Friston et al., 1996; Yan et al., 2013). We applied nuisance regression both with and without global signal regression (GSR) to examine the robustness of our findings against different preprocessing procedures. Though GSR causes a reduction of positive connectivity and an increase of negative connectivity for single condition (Murphy et al., 2009), the effects might be counteracted during the comparison between conditions and thus would not be obvious for the between-condition results that we were more interested in. In the current study, we utilized the DWI data to verify the positions of LGN seed regions for each subject (see below). The preprocessing steps for the DWI data included skull stripping and eddy current correction, both of which were implemented in FMRIB Software Library (FSL)^3^.

### Seed Based FC Analyses

The seed regions of LGN (left LGN: 230 mm^3^, right LGN: 228 mm^3^) and Pu (left PU: 2385 mm^3^, right PU: 2380 mm^3^) were defined from Morel’s thalamus atlas (Morel et al., 1997; Krauth et al., 2010) with resolution of 1 mm. As bilateral LGN are relatively small, their positions may be shifted during spatial normalization due to inter-individual differences. To verify the accuracy of their positions, we performed the probabilistic fiber tracking from the LGN to V1 for each subject by using FMRIB Diffusion Toolbox (FDT)^4^. The distributions on diffusion parameters at each voxel were calculated for probabilistic tractography and the LGN regions (after being transformed into the diffusion space) were chosen as the seed masks for the probabilistic tractography with default parameters of 5000 streamline samples, a curvature threshold of 0.2, maximum number of steps of 2000 and a step length of 0.5 mm.

**FIGURE 1:**
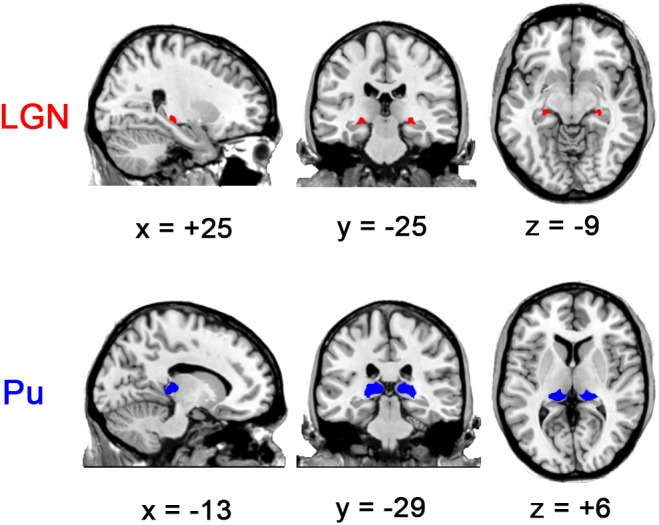
Visual thalamic nuclei ROIs defined using Morel’s thalamus atlas. Note that the Pu ROIs were eroded by 1 mm. The left side of all the figures in this study corresponds to the left hemisphere of the brain.

Although animal studies suggested the functional divergence of Pu subnuclei (Cappe et al., 2009; Bridge et al., 2016), a clear description of their functional roles, especially in humans, is still lacking. The boundary of these subdivisions might also be obscured in 3T images. Thus the four parts of Pu from the atlas [medial Pu (PuM), inferior Pu (PuI), lateral Pu (PuL) and anterior Pu (PuA)] were merged into a whole Pu ROI for each hemisphere, which were then eroded by 1 mm (Figure 1) to reduce influences from neighboring voxels. Notably, the erosion was not done for LGN ROIs because of their small sizes. Next, both the Pu and LGN ROIs were downsampled to 3 mm^3^ × 3 mm^3^ × 3 mm^3^ to match the resolution of EPI data, resulting in 42 voxels (1134 mm^3^) and 41 voxels (1107 mm^3^) for Pu ROIs (left and right, respectively) and 10 voxels (270 mm^3^) for both left and right LGN. We then calculated the FC between the mean BOLD time course of each seed (i.e., left LGN, right LGN, left Pu, and right Pu) with all the voxels in the brain using Pearson’s correlation. The correlation coefficients were then converted to *z*-values using Fisher’s r-to-z transformation.

### ALFF Calculation

To examine possible links between the thalamic FC and the known EO-EC ALFF differences, we calculated ALFF additionally in this work. In particular, the time series were transformed into the frequency domain via fast Fourier transform (FFT). Then, the square root of the obtained power spectrum was averaged across the low-frequency band (0.01–0.1 Hz) for each voxel. Individual ALFF maps were divided by the global mean, as was performed in the PET study (Raichle et al., 2001).

### Statistical Analyses

Both the within-condition patterns (only for FC) and the between-condition differences were analyzed with the framework of general linear model, using the non-parametric permutation tests as implemented in Permutation Analysis of Linear Models (PALM, 10000 permutations) (Winkler et al., 2016). The cluster-defining threshold was two-tailed *p* < 0.01, and the family-wise error (FWE) corrected *P*-values were maintained at a threshold of two-tailed *p* < 0.05.

### Association Between the Thalamic FC and ALFF Results

To test the association between thalamocortical interaction and the previously observed EO-EC ALFF differences, we firstly examined the overlap of the EO-EC differences in the two indices. Then, we calculated the correlation between the averaged changes of FC values and those of ALFF across subjects within the overlapping regions. The procedures were applied on each seed separately.

## Results

### Whole Brain FC of LGN and Pu

**FIGURE 2:**
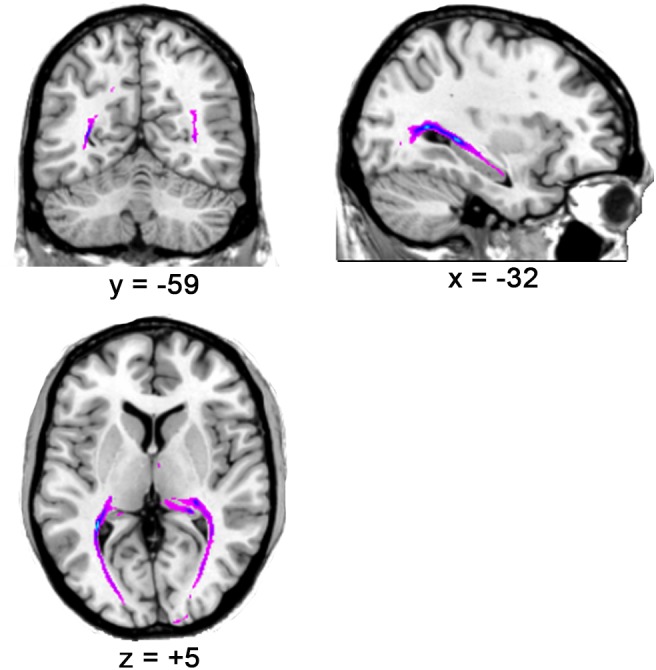
The optic radiation tracking result for one representative subject.

The results showed that the optic radiation (from LGN to V1) could be tracked in all subjects (please see Figure 2 for the tracking result for one representative subject and the Supplementary Video S1 for all subjects), thereby suggesting the accuracy of LGN positions in all subjects.

**FIGURE 3:**
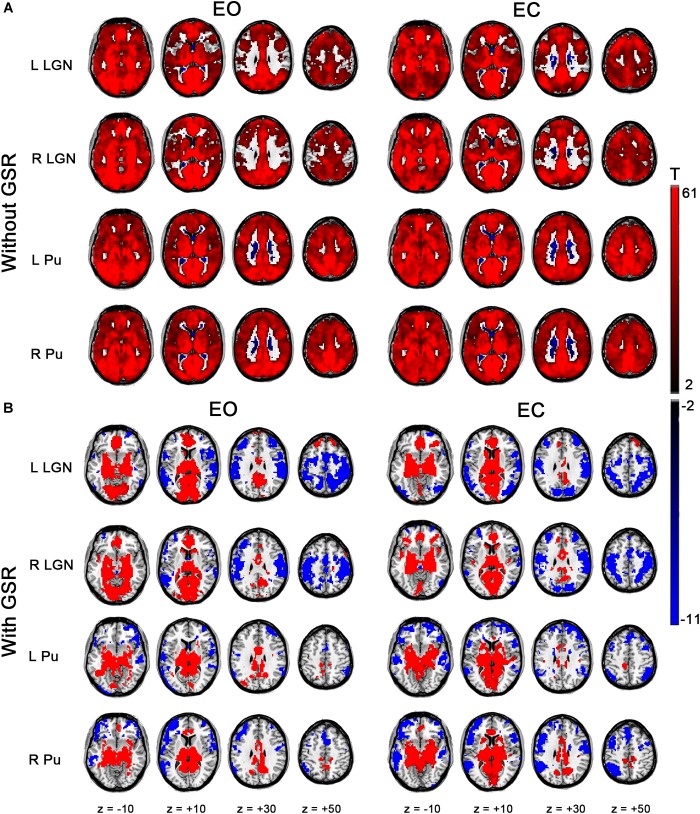
Within-condition statistical results of FC for bilateral LGN and Pu. **(A)** Results for EO and EC states without GSR. **(B)** Results with GSR (*p* < 0.05, cluster-level FWE corrected).

Each ROI showed strong positive correlation with almost all the brain areas in EO and EC (Figure 3A, *p* < 0.05, corrected) for the results without GSR. After GSR, positive correlation was detected in the entire thalamus, cingulate gyrus, middle frontal gyrus (MFG), insula and visual cortex; and negative correlation emerged in precuneus, superior parietal gyrus (SPG), primary sensorimotor cortex (PSMC), a large portion of supplementary motor area (SMA), superior temporal gyrus (STG), superior frontal gyrus (SFG), and MFG in both conditions for LGN. Both left and right Pu showed positive correlation with thalamus, part of precuneus and visual cortex, and negative correlation with angular gyrus, STG, middle temporal gyrus (MTG), SFG and MFG.

### EO-EC Differences in the Thalamic FC

**FIGURE 4:**
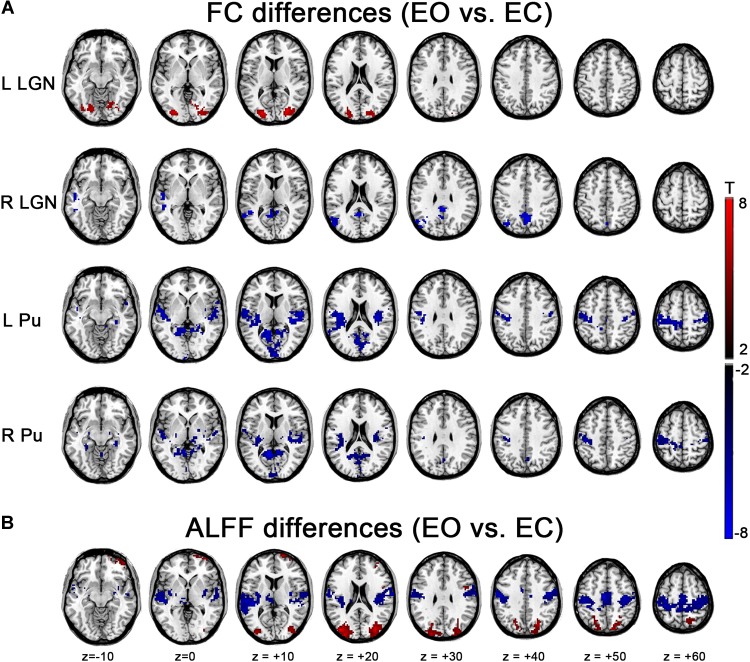
Effects of EO/EC on thalamic FC and ALFF. **(A)** Between-condition differences in the thalamic FC. **(B)** Between-condition differences in ALFF. The results were based on the data analyzed without GSR. Red and blue regions indicated increased and decreased FC (or ALFF) in EO compared with EC, respectively (*p* < 0.05, cluster-level FWE corrected).

The left LGN had an increased (i.e., EO > EC) FC with middle occipital gyrus (MOG), superior occipital gyrus (SOG), inferior occipital gyrus (IOG), fusiform gyrus and lingual gyrus. Decreases (i.e., EO < EC) were found within a few voxels in the left PSMC and left Heschl’s gyrus during EO compared to during EC. The right LGN exhibited a decreased FC in the left MTG and precuneus (Figure 4A, *p* < 0.05, corrected). For Pu, decreases were mainly in the left PSMC, right parahippocampal gyrus, calcarine, lingual gyrus and a few voxels in the thalamus, except for a broader connection with the STG, rolandic region, and the PSMC for the left Pu (Figure 4A, *p* < 0.05, corrected). Though GSR had prominent effect on FC results of single state (Figure 3), the differences in FC based on GSR showed similar patterns with those without GSR, except that the right LGN only exhibited increased FC in the extrastriate cortex with GSR (Supplementary Figure S1A, *p* < 0.05, corrected) and the right PU only exhibited decreased FC in the PSMC and temporal cortex without GSR (Figure 4A, *p* < 0.05, corrected).

### EO-EC Differences in ALFF

Significantly higher ALFF were found in bilateral MOG and SOG, while lower ALFF occurred in the SMA, bilateral PSMC and auditory areas, including Heschl’s gyrus and STG during EO compared to that of EC. The pattern was almost consistent with our previous findings (Liu D. et al., 2013), irrespective of GSR (Figure 4B and Supplementary Figure S1B, *p* < 0.05, corrected).

### Overlap and Correlation Between ALFF and FC Based EO-EC Differences

**FIGURE 5:**
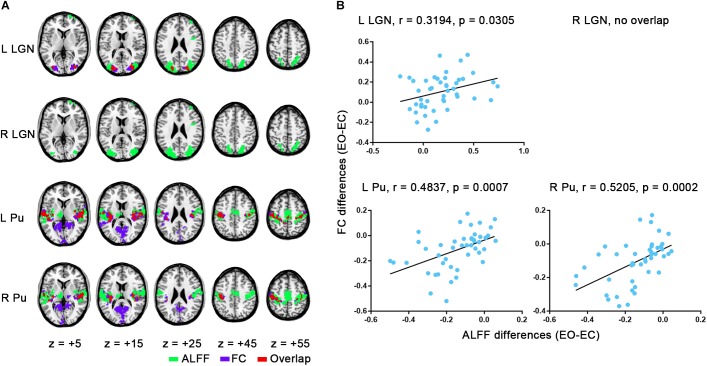
Overlap maps and correlation results. **(A)** Overlap of group-level FC differences for each seed with ALFF comparisons (EO-EC). **(B)** Correlation between averaged changes of FC and changes of ALFF values within the overlapping region across subjects. The results were based on the data analyzed without GSR.

Interestingly, the regions with increased ALFF overlapped to a considerable extent with the LGN’s FC differences, while the regions with decreased ALFF showed a large overlap with the FC differences of Pu, although between-hemispheric discrepancy existed (Figure 5A). Further verification comes from a correlation analyses in which significant correlation was observed, except for the right LGN (Figure 5B, left LGN: *r* = 0.3194, *p* = 0.0305; right LGN: no overlap; left Pu: *r* = 0.4837, *p* = 0.0007; right Pu: *r* = 0.5205, *p* = 0.0002). For results with GSR, the correlation was weaker, although all of them showed considerable overlap except for right Pu (Supplementary Figure S2). For all the aforementioned analyses, the results without spatial smoothing showed similar patterns (Supplementary Figures S3–S7), except that good consistency was observed between the results of bilateral LGN, irrespective of GSR (Supplementary Figures S4A, S6A, *p* < 0.05, corrected).

## Discussion

One critical step toward understanding the observed ALFF differences across resting state conditions (EO versus EC) (McAvoy et al., 2008; Yan et al., 2009; Jao et al., 2013; Liu D. et al., 2013; Yuan et al., 2014; Zou et al., 2015; Zhang et al., 2018) is to depict the relationship between the thalamus and ALFF modulation. LGN and Pu represent important thalamic relay nuclei related to visual processing. Herein we found that LGN and Pu exhibited significantly different thalamic FC across conditions, which were highly associated with the changes of ALFF in several sensory networks between EO and EC.

### FC Between LGN and Extrastriate Cortex, but Not PVC, Was Modulated

Although the FC results of the single condition showed largely similar patterns, there were notable FC differences between EO and EC both for LGN and Pu. The increased FC for the left LGN was mainly located in the MOG, SOG, and IOG, which belong to the lateral visual network. The right LGN showed more similar results with the left LGN if GSR was applied (Supplementary Figure S1A) or spatial smoothing was not performed (Supplementary Figures S4A, S6A). The right LGN is slightly smaller than the left, thus may be more vulnerable to the spatial blurring effects. Considering that no marked hemispherical asymmetry has been reported concerning LGN functions, we believe in general accordance between the hemispheres for the FC results of LGN.

Modulation of the LGN’s FC was only significant in the lateral visual network but not in the medial visual network, although it is well known that LGN relays the main visual input from the retina to PVC and tends to be co-activated with PVC in visual task (Kastner et al., 2006). One possible explanation is that, task evoked activity and spontaneous activity both in LGN and PVC occur at different temporal scales. Task activation is usually assumed to reflect rapid changes of neural activities whereas spontaneous activity was found to be more related to the slow (<0.1 Hz) modulation of the band-limited power brain signal (Leopold et al., 2003; He et al., 2008; Nir et al., 2008; Liu et al., 2010). For BOLD fMRI, FC metrics may not be sensitive enough to capture the changes of synchronization of rapid activities due to the sluggish nature of neuro-vascular coupling. Although it has been shown that PVC is significantly correlated with LGN via low-frequency fluctuations (Fox et al., 2009), their correlation is more likely to reflect the core functional architecture around which task-related synchronization occurs (Cole et al., 2014; Krienen et al., 2014). For lateral visual network, however, its increased FC with LGN during EO might reflect the strengthened slow modulation of the intrinsic activity which may encode the improved visual awareness. Thalamic relay cells often exhibit dual response modes (Sherman, 1996, 2001). Tonic firing is characterized by continuous stream of action potentials whereas burst firing is made of clusters of action potentials with silent periods about tens of miliseconds. The former conveys information more linearly so that it is suited to high-fidelity information passing, whereas the latter is more efficient in stimuli detection (Sherman, 1996, 2001). On the other hand, increasing evidence has suggested the existence of a shortcut between the LGN and the extrastriate cortex, which is capable of facilitating visual responses in some instances. For example, in two macaque monkeys with chronic PVC lesions, Schmid et al. (2010) found direct LGN projections to the extrastriate cortex that supported blindsight, and these PVC-independent visual functions were abolished once with temporary LGN inactivation (Schmid et al., 2010). Support also comes from an observation that visual response latencies in extrastriate visual areas were as short as in the PVC (Schmolesky et al., 1998). The extrastriate cortex has been found to be deactivated in a somatosensory task, in which the authors interpreted it as “closing the mind’s eye” (Kawashima et al., 1995). Taken together, the LGN might interact with the extrastriate cortex to prepare for incoming visual stimuli when the eyes are open, and synchronization of slow activity within the thalamocortical system may play important roles.

### FC of Pu With Other Sensory Modalities Beyond Visual Areas

Largely distinct from the results of LGN, the FC of Pu was primarily decreased under EO than EC in non-visual modalities including the auditory cortex and PSMC. The trends were stronger for cases without GSR than those with GSR (Figure 4A and Supplementary Figure S1A). Actually, Pu’s FC has been compared across resting state conditions, but the comparison was only focused on the visual cortex (Zou et al., 2009). Although we extended the findings to the whole brain, our results are controversial in the visual cortex with that study. Zou et al. (2009) found significantly increased FC in the visual area during EO than during EC for the right Pu and no differences for left Pu. In the current study, we did not detect any significant changes in the visual cortex for the results with GSR (Note: as Zou et al. (2009) only reported Pu’s results with GSR, here we compared our results based on GSR with theirs). The discrepancy may be due to different statistical approaches or thresholds we used. Since Eklund et al. (2016) reported that parametric statistical approach with clusterwise inference could lead to a high degree of false positives, here we tended to believe that EO/EC had little significant effect on Pu’s FC within visual areas if GSR was done. Additionally, the discrepant preprocessing methods (e.g., head motion regression, Friston et al., 1996; Yan et al., 2013) may account for the inconsistency. The observation that Pu had weaker FC with auditory and somatosensory regions during EO than EC coincides with the functional role of Pu in multisensory interplay (Cappe et al., 2009). It has been reported that neurons in PuM can respond to both visual (Gattass et al., 1979) and auditory (Yirmiya and Hocherman, 1987) stimuli. Some findings further demonstrated that the lesion of Pu, as well as the right frontal lobe, might result in auditory attentional neglect (Hugdahl et al., 1991).

### Association Between Thalamus and Modulation of Cortical ALFF in Sensory Regions

It has been consistently reported that EO/EC has significant influences on variability of spontaneous brain activity in sensory regions beyond V1, including PSMC, PAC and extrastriate cortex (Yan et al., 2009; Liu D. et al., 2013; Yuan et al., 2014; Zou et al., 2015; Zhang et al., 2018). These sensory regions cannot be detected to be active in those studies where the mean activities [e.g., regional cerebral blood flow (rCBF) in arterial spin labeling (ASL) (Zou et al., 2015) or activation in BOLD fMRI (Zhang et al., 2018)] across a prolonged period of time were assessed and compared across conditions. For the EO-EC contrast, these findings suggest that the variability of brain activity may represent a novel response mode that is distinct from traditional activation.

The mechanisms are still unclear. In the present study, we found the ALFF differences between EO and EC had considerable overlap with the impacts of EO/EC on FC of LGN and Pu. Namely, the regions with increases in ALFF (i.e., EO > EC) overlapped to a considerable extent with the LGN’s FC differences, while those with decreased ALFF (i.e., EO < EC) showed a large overlap with FC differences of Pu. Moreover, significant correlation between them was detected for the data without GSR. A similar trend was also obtained in the data with GSR, though the correlation was not significant. The weakened correlation could be due to the distortion effect of GSR on time series, since GSR can bring an artificial negative correlation between brain regions (Murphy et al., 2009; Saad et al., 2012). Here, based on the overall consistency of overlap results between data with and without GSR (xFigure 5 and Supplementary Figures S2, S5, S7), we believe in a significant relationship between the thalamic FC and cortical ALFF modulation.

Although the causal relationship is still unknown, we provided empirical evidences that the EO/EC modulation of the spontaneous and low-frequency fluctuations in the sensory regions beyond V1 had a close relationship with the thalamus. The dissociation between EO/EC modulation on ALFF and EO-EC activation might be related with distinct physiological properties of thalamus, such as different firing modes of thalamus relay cells (Sherman, 1996, 2001). In the future, studies about their relationships will benefit greatly from improvements in acquisition techniques of BOLD fMRI (Hale et al., 2010; Van Essen et al., 2012; Raemaekers et al., 2014) and temporal analysis approaches (Liu X. et al., 2013; van Oort et al., 2018). The studies could illuminate how the thalamus generates the two distinct forms of neural signals (i.e., activation and modulation of ALFF). Given that the dual response modes are not unique to visual thalamic nuclei but a general property of thalamic relay cells (Sherman, 2001) and thalamus has widespread connections with the entire cortex (Sherman and Guillery, 2006; Sherman, 2007), investigations into its relationship with activation/ALFF modulation distinction would be informative for mechanisms of regional spontaneous fluctuations of human brain in other systems (Garrett et al., 2011, 2013, 2014).

## Conclusion

In conclusion, we revealed an association between modulation of ALFF and that of the thalamic FC in sensory systems. Our findings supported the participation of the thalamus in amplitude modulation of low-frequency spontaneous fluctuations in sensory networks, and highlighted the significance of thalamus in understanding the nature of regional spontaneous brain activity.

## Ethics Statement

In this manuscript, we have used the public resting state fMRI dataset available at the NITRC (http://fcon_1000.projects.nitrc.org/indi/IndiPro.html, Beijing: eyes open and eyes closed study). It was approved by the ethics committee of Institutional Review Board of Beijing Normal University Imaging Center for Brain Research. Before the data was collected, this study was approved by the ethics committee of the Institutional Review Board of the Beijing Normal University, Imaging Center for Brain Research. Written informed consent was obtained from each participant.

## Author Contributions

D-QL designed the study. SQ, XW, and XQ analyzed the data. All authors wrote the manuscript.

## Conflict of Interest Statement

The authors declare that the research was conducted in the absence of any commercial or financial relationships that could be construed as a potential conflict of interest.

## Footnotes

1 http://fcon_1000.projects.nitrc.org/indi/IndiPro.html

2 http://rfmri.org/DPABI

3 www.fmrib.ox.ac.uk/fsl

4 www.fmrib.ox.ac.uk/fsl/fdt
