# Supplementary material for: Links Between the Amplitude Modulation of Low-Frequency Spontaneous Fluctuation Across Resting State Conditions and Thalamic Functional Connectivity
Source: Front Hum Neurosci. 2019 Jun 13;13:199. doi: 10.3389/fnhum.2019.00199 (PMC6584839; doi:10.3389/fnhum.2019.00199)
Supplement: Supplementary file 1 [file Data_Sheet_1.doc]

**Supplementary Figure 1. Effects of EO/EC on thalamic FC and ALFF.** (A) Between-condition differences in the thalamic FC. (B) Between-condition differences in ALFF. The results were based on the data analyzed **with GSR**. Red and blue regions indicated increased and decreased FC (or ALFF) in EO compared with EC, respectively (p < 0.05, cluster-level FWE corrected).

**
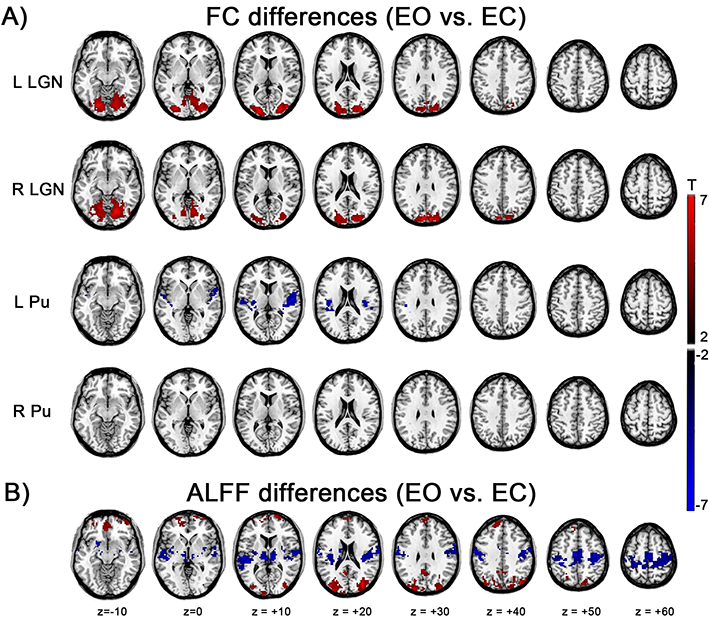
**

**Supplementary Figure 2. Overlap maps and correlation results.** (A) Overlap of group-level FC differences for each seed with ALFF comparisons (EO-EC). (B) Correlation between averaged changes of FC and changes of ALFF values within the overlapping region across subjects. The results were based on the data analyzed **with GSR**.

**
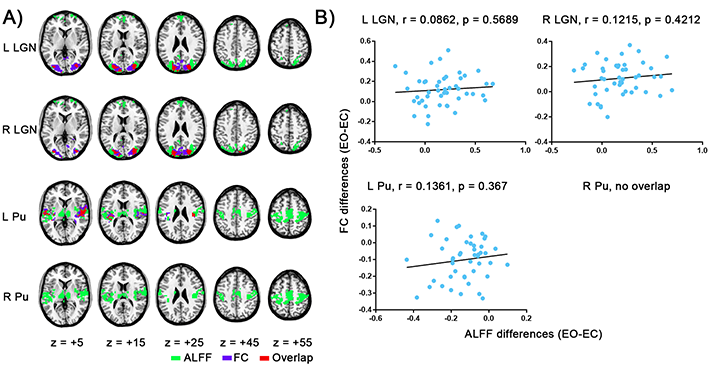
**

**Supplementary Figure 3. Within-condition statistical results of FC for bilateral LGN and Pu.** (A) Results for EO and EC states without GSR. (B) Results with GSR (p < 0.05, cluster-level FWE corrected). The results were based on the data analyzed **without** **spatial smoothing**.

**
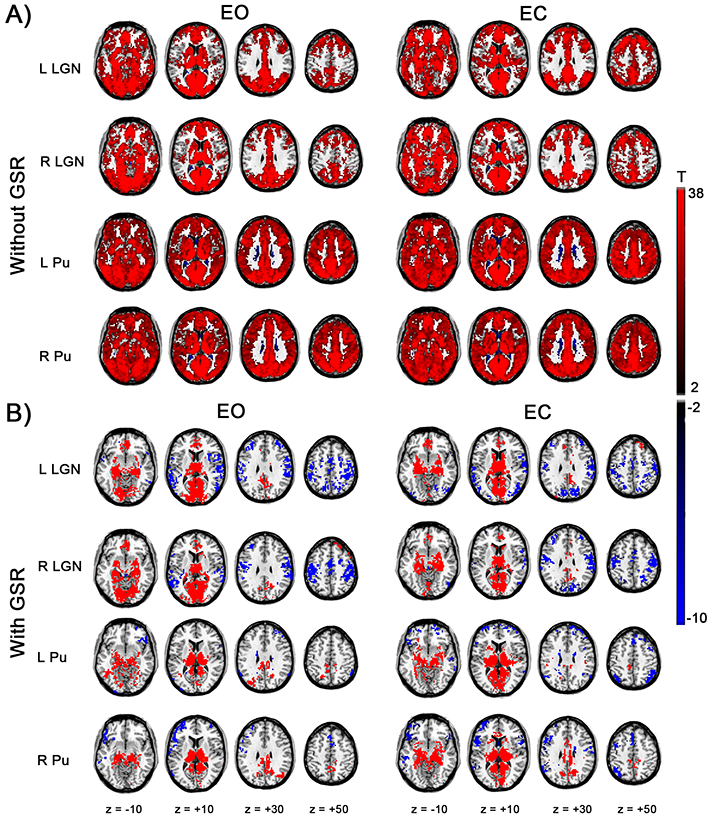
**

**Supplementary Figure 4. Effects of EO/EC on thalamic FC and ALFF.** (A) Between-condition differences in the thalamic FC. (B) Between-condition differences in ALFF. The results were based on the data analyzed **without GSR or spatial smoothing**. Red and blue regions indicated increased and decreased FC (or ALFF) in EO compared with EC, respectively (p < 0.05, cluster-level FWE corrected).

**
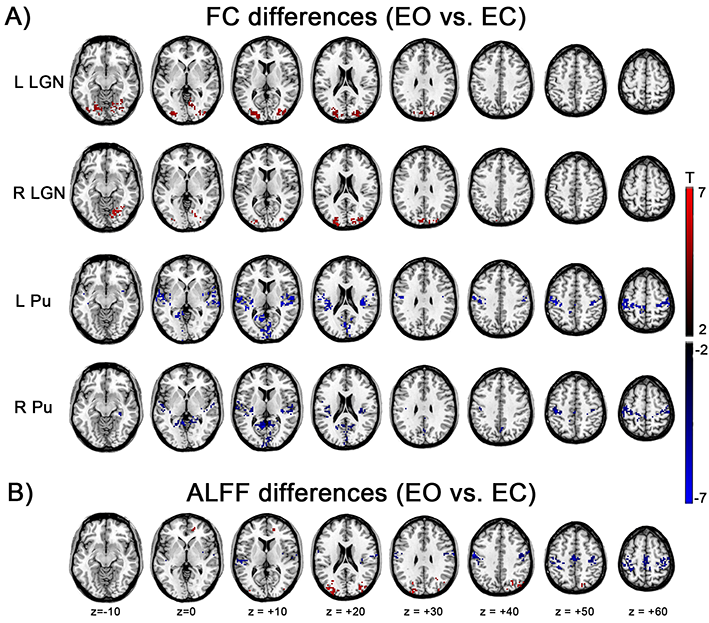
**

**Supplementary Figure 5. Overlap maps and correlation results.** (A) Overlap of group-level FC differences for each seed with ALFF comparisons (EO-EC). (B) Correlation between averaged changes of FC and changes of ALFF values within the overlapping region across subjects. The results were based on the data analyzed **without GSR or spatial smoothing**.

**
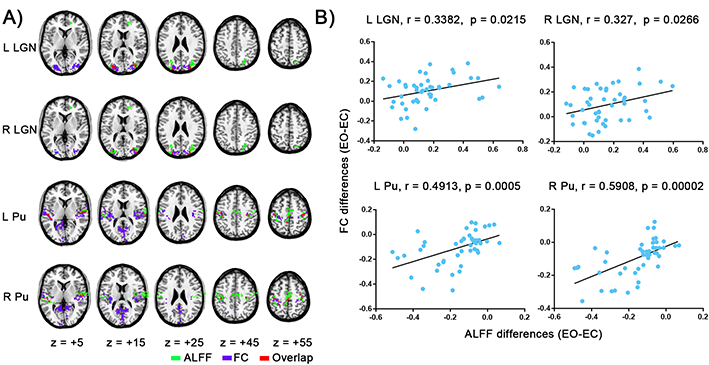
**

**Supplementary Figure 6. Effects of EO/EC on thalamic FC and ALFF.** (A) Between-condition differences in the thalamic FC. (B) Between-condition differences in ALFF. The results were based on the data analyzed **with GSR and without spatial smoothing**. Red and blue regions indicated increased and decreased FC (or ALFF) in EO compared with EC, respectively (p < 0.05, cluster-level FWE corrected).

**
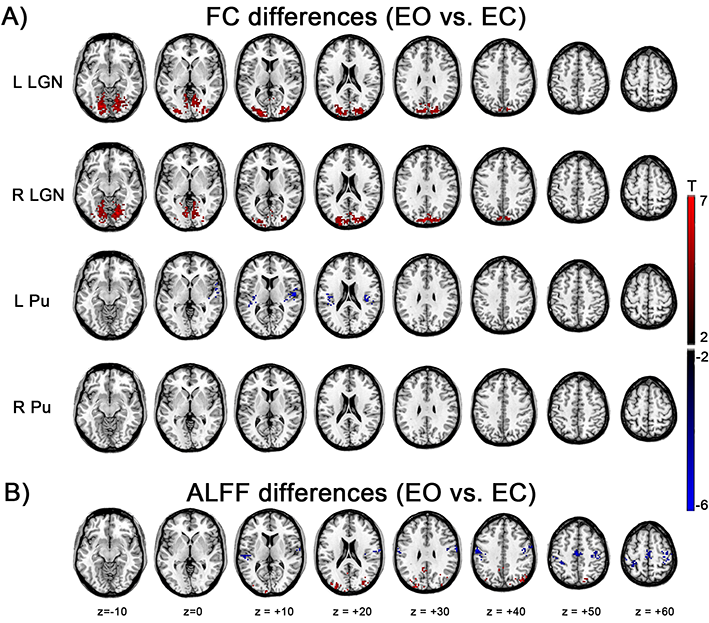
**

**Supplementary Figure 7. Overlap maps and correlation results.** (A) Overlap of group-level FC differences for each seed with ALFF comparisons (EO-EC). (B) Correlation between averaged changes of FC and changes of ALFF values within the overlapping region across subjects. The results were based on the data analyzed **with GSR and without spatial smoothing**.

**
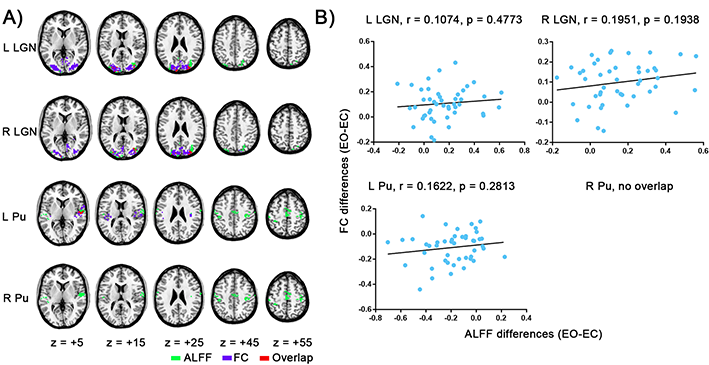
**

**Video 1. Probabilistic fiber tracking results of LGN for all subjects.** Purple and blue regions indicated the tracked fibers seeded from the bilateral LGN ROIs.
